# Supplementary material for: Positive Association between the Use of Quinolones in Food Animals and the Prevalence of Fluoroquinolone Resistance in E. coli and K. pneumoniae, A. baumannii and P. aeruginosa: A Global Ecological Analysis
Source: Antibiotics (Basel). 2021 Oct 1;10(10):1193. doi: 10.3390/antibiotics10101193 (PMC8532820; doi:10.3390/antibiotics10101193)
Supplement: Supplementary file 1 [file antibiotics-10-01193-s001.zip › antibiotics-1396412-supplementary.pdf]

Online supplementary file

Box S1. Details for how IQVIA calculates country-level fluoroquinolone consumption estimates.

IQVIA uses national sample surveys that are performed by pharmaceutical sales distribution channels to estimate antimicrobial consumption from the volume of antibiotics sold in retail and hospital pharmacies. The sales estimates from this sample are projected with use of an algorithm developed by IQVIA to approximate total volumes for sales and consumption. Quinolone consumption (Moxifloxacin, Ciprofloxacin, Gemifloxacin, Ofloxacin, Levofloxacin, Lomefloxacin, Norfloxacin, Enoxacin, Gatifloxacin, Trovafloxacin, Sparfloxacin) estimates are reported as the number of standard doses (a dose is classified as a pill, capsule, or ampoule) per 1000 population per year [1].

Table S1. Sources used by CDDEP to provide antimicrobial resistance prevalence estimates. Results are provided by country.[2]

Country	Data Source
**Argentina**	WHONET-Argentina Network and SIREVAII-Argentina Network
**Australia**	Australian Group of Antimicrobial Resistance (AGAR)
**Austria, Belgium, Bulgaria, Croatia, Cyprus, Czech Republic, Denmark, Estonia, Finland, France, Germany, Greece, Hungary, Iceland, Ireland, Italy, Latvia, Lithuania, Luxembourg, Malta, Netherlands, Norway, Poland, Portugal, Romania, Slovakia, Slovenia, Spain, Sweden, United Kingdom**	European Antimicrobial Resistance Surveillance Network (EARS-Net)
**Bahrain, Brazil, Cambodia, Egypt, Ethiopia, Indonesia, Iran, Japan, Jordan, Kosovo, Laos, Lebanon, Madagascar, Mali, Myanmar, Nepal, Nigeria, Oman, Philippines, Republic of Korea, Saudi Arabia, Sudan, Thailand, Tunisia, United Arab Emirates, Zambia**	Global Antimicrobial Resistance Surveillance System (GLASS)
**Belarus, Bosnia and Herzegovina, Georgia, Kosovo, Former Yugoslav Republic of Macedonia, Montenegro, Russia, Serbia, Switzerland, Turkey, Ukraine**	Central Asian and European Surveillance of Antimicrobial Resistance Network (CAESAR)

**Canada**	Canadian Antimicrobial Resistance Alliance (CARA)
**Chile**	Chilean Society of Infectious Diseases
**China**	CHINET surveillance
**Ecuador**	Reference Laboratory for Antimicrobial Resistance at the National Institute of Public Health Research (INSPI)
**Ghana, Zimbabwe**	Lancet Laboratories Pvt. Ltd
**India**	SRL Diagnostics
**Kenya**	Private tertiary hospital
**Malawi**	Queen Elizabeth Central Hospital
**Malaysia**	National Surveillance of Antimicrobial Resistance, Malaysia
**Mexico**	Hospital Civil de Guadalajara "Fray Antonio Alcalde"
**New Zealand**	Public Health Surveillance
**Pakistan**	Chugtai's Laboratory Private Ltd.
**South Africa**	South Africa Society for Clinical Microbiology
**Taiwan**	Taiwan Nosocomial Infection Surveillance (TNIS)
**United States of America**	The Surveillance Network (TSN)
**Venezuela**	Programa Venezolano de Vigilancia de la Resistencia a los Antimicrobianos (PROVENRA)
**Vietnam**	Viet Nam Resistance (VINARES) Project

Table S2. Sensitivity analysis of Table 2. Linear regression models testing the country-level association between quinolone consumption in food-producing animals and humans and the prevalence of fluoroquinolone resistance in *E. coli, K. pneumoniae, A. baumanii* and *P. aeruginosa* spp. ***in all countries excluding China*** [coefficients (95% confidence intervals)]

	E. coli			***K. pneumoniae***			***A. baumanii***			***P. aeruginosa***		
	Model 1	Model 2	Model 3	Model 1	Model 2	Model 3	Model 1	Model 2	Model 3	Model 1	Model 2	Model 3
Quinolones food animals	11.8 (-1.9-25.6)	-	1.2 (-11.3-13.8)	21.3 (-11.9-43.7)	-	12.9 (-11.8-37.8)	51.2 (19.9-82.4)**	-	32.7 (5.8-59.6)*	12.8 (19.1-23.7)*	-	7.5 (-3.9-19.1)
Quinolones humans	-	.02 (.01-.03)**	.02 (.01-.04)**	-	.03 (.01-.04)**	.02 (-.004-.04)	-	.05 (.02-.08)**	.04 (.02-.07)**	-	.02 (.01-.03)**	.01 (.00-.02)*
N	34	46	32	30	41	31	25	34	24	28	36	27
R^2	0.08	0.33	0.41	0.12	0.20	0.21	0.33	0.29	0.59	0.18	0.28	0.28

* P-value <0.05, ** P-value <0.005

Table S3. Sensitivity analysis of Table 2. Spearman's correlation matrix of country-level prevalence of fluoroquinolone resistance (%) in 4 bacterial species and quinolone consumption in food-producing animals and fluoroquinolone consumption in humans ***in all countries excluding China***

	Acinetobacter baumannii	*Escherichia coli*	*Pseudomonas aeruginosa*	*Klebsiella pneumoniae*	*Food-animal FQ consumption*	**Human FQ consumption**
Acinetobacter baumannii	1					
Escherichia coli	0.67**	1				
Pseudomonas aeruginosa	0.77**	0.78**	1			
Klebsiella pneumoniae	0.73**	0.63**	0.91**	1		
Food-animal FQ consumption	0.49*	0.52**	0.55**	0.60**	1	
**Human FQ consumption**	0.58**	0.62**	0.56**	0.43*	0.42*	1

* P<0.05 ** P<0.005; FQ - fluoroquinolone

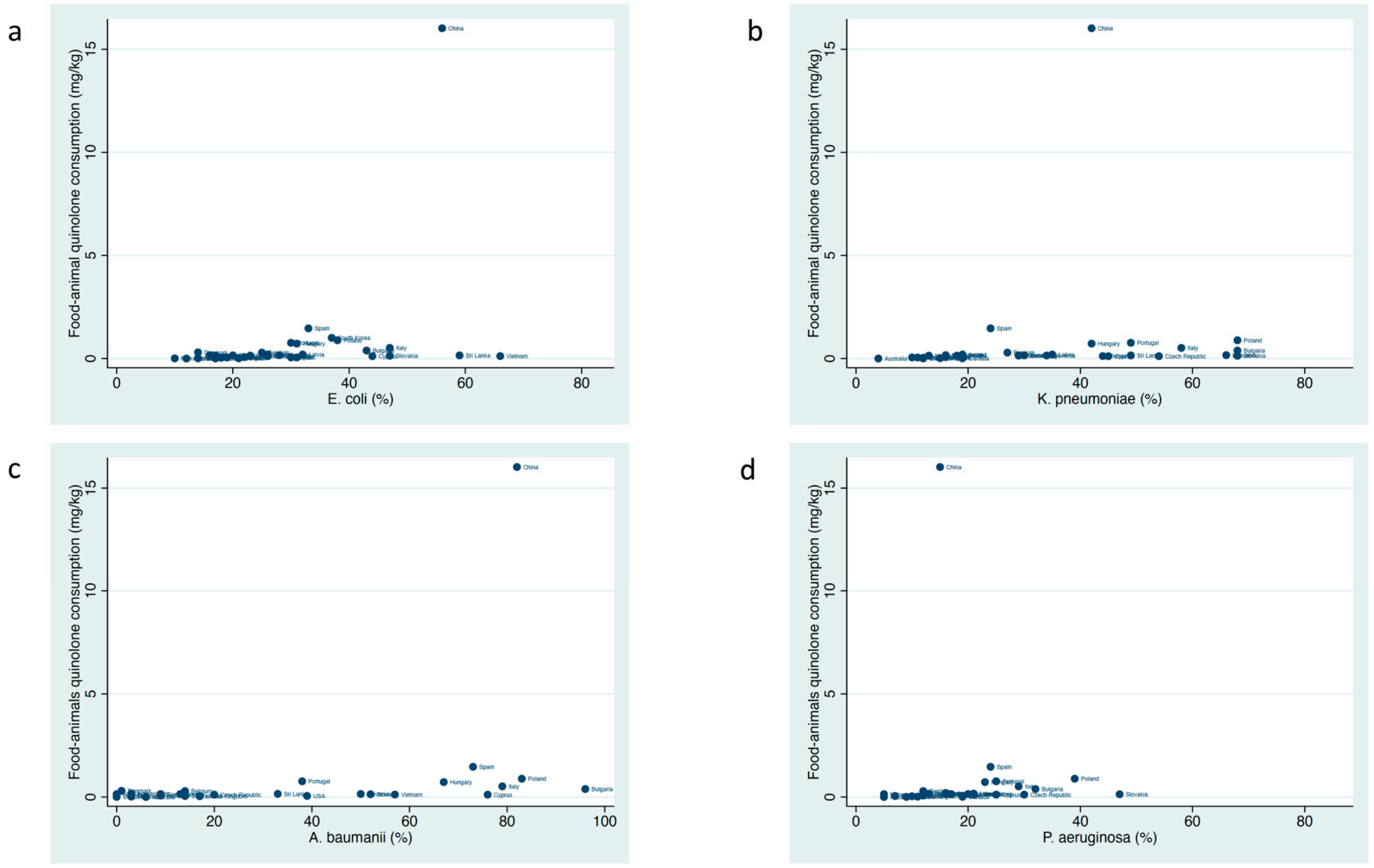

Figure S1. Scatter plots of the country-level association between quinolone consumption in food-producing animals and the prevalence of fluoroquinolone resistance in a) *E. coli*, b) *K. pneumoniae,* c) *A. baumanii* and d) *P. aeruginosa* spp.

References

1. Kenyon C, Buyze J, Wi T. Antimicrobial consumption and susceptibility of Neisseria gonorrhoeae: a global ecological analysis. Frontiers in medicine. 2018;5:329.

2. The Center for Disease Dynamics Economics & Policy. ResistanceMap: Antibiotic resistance. 2021 [cited 2021]. Available from: 2021. https://resistancemap.cddep.org/AntibioticResistance.php.
